# Effect of binding immunoglobulin protein on induction of regulatory B cells with killer phenotype during inflammation and disease

**DOI:** 10.4155/fsoa-2018-0121

**Published:** 2019-03-05

**Authors:** Bongani Motaung, Andre G Loxton

**Affiliations:** 1 DST/NRF Centre of Excellence for Biomedical Tuberculosis Research, Stellenbosch University, Cape Town, South Africa; PO Box 241, Cape Town 8000, South Africa; 2 South African Medical Research Council Centre for Tuberculosis Research, Stellenbosch University, Cape Town, South Africa; PO Box 241, Cape Town 8000, South Africa; 3 Division of Molecular Biology & Human Genetics, Faculty of Medicine & Health Sciences, Stellenbosch University, Cape Town, South Africa; PO Box 241, Cape Town 8000, South Africa

**Keywords:** apoptosis, autoimmune disease, binding immunoglobulin protein, chronic disease, immune response, *Mycobacterium tuberculosis* infection, necrosis and inflammation, regulatory B cells, UPR

## Abstract

Immune responses result from different immune cells acting in synergy to successfully fight infections. This requires a high degree of regulation to prevent excessive production of inflammatory products leading to other disease forms. Regulatory B cells are classified based on surface immunoglobulin expression. These cells are reported to resolve inflammation during chronic or autoimmune diseases. However, during chronic inflammation, their frequencies have been shown to be affected, and they can be induced by exposure to extracellular binding immunoglobulin protein (BiP). This review focuses on the effects on immune cells by extracellular or secreted BiP during various chronic inflammatory responses. For example, cell stress associated with *Mycobacterium tuberculosis* infection leads to accumulation of unfolded proteins that subsequently activate BiP and its three signal transducers intracellularly. Furthermore, BiP can be translocated from the endoplasmic reticulum to the extracellular environment where it binds immune cells as an autoantigen and leads to functional changes.

## Lay abstract

Immune responses during tuberculosis disease require balanced cell interactions. These include antigen-presenting cells, effector cells and regulatory cells. B lymphocytes can mediate regulatory function during chronic diseases and lead to better disease outcome. These specialized cells mediate this function through both surface and soluble protein expression. Their development can be facilitated by different stimuli including binding immunoglobulin protein. This protein resides in the endoplasmic reticulum where it functions in proper protein folding; however, it can escape this location to the extracellular phase, where it affects immune cell function leading to development of regulatory traits on B cells.

## Immunity, B cells & regulatory B cell responses during inflammation (autoimmune/infection)

Immunological studies have shown that successful clearance of any invading pathogen depends on effective balance between immune cells and their secreted products such as cytokines, antibodies and chemokines. Depending on the nature of infection, immune cell balance can be altered through biological processes such as necrosis, pyroptosis, programmed cell death and apoptosis [1]. These cellular processes are triggered mostly by intracellular pathogens such as *Mycobacterium tuberculosis*, which has evolved to suppress immune responses by effector T cells through release of bacterial vesicles that expresses lipoarabinomannan and other lipoglycans [2]. These bacteria suppress immune activation through recruitment of mesenchymal stem cells, which secrete immunosuppressive cytokines and nitric oxide [3].

Even though the Bacillus Calmette-Guerin (BCG) vaccination has been in use for years as it stimulates the immune system and shortens the specific antibody response against *M. tuberculosis* infection, which targets lipoarabinomannan embedded on their cell wall [4], there is still a need for advances that will better eradiate or control the infection. These antibodies are secreted by a subpopulation of B cells (plasma cells). Furthermore, they facilitate rapid cell-mediated immunity through pathogen opsonization and binding of their Fcγ receptors (FcγR) with professional antigen-presenting cells (APC) that result in internalization of the pathogen [5]. However, *M. tuberculosis* is known to reside and multiply within these antigen-presenting cells, leading to formation of granuloma structures [6,7]. Dissemination of these structures and progression to active tuberculosis has been shown to affect the frequency of immunological cells such as circulating peripheral B cells [8,9]. The tuberculosis (TB) pathogen takes advantage of this imbalance in the immune system and multiplies further, thus infecting more and more cells.

Immune system inadequacy or manipulation by *M. tuberculosis* has highlighted the importance of exploring other functions played by immune cell subtypes as a means to better control infection. It has become evident through research that regulatory functions in different immunological cells, including B cells, play more than just a role of suppressing aggressive immune responses during autoimmune and infectious diseases. These regulatory subsets play a major role in balancing the immune system and better facilitate elimination and control of pathogens and resolution of inflammation [10–13]. Immune suppression functions are mediated by a group of specialized regulatory cells in the innate (myeloid-derived suppressor cells and natural killer cells) [14,15] and adaptive arms, mainly of the T (regulatory T cells [Tregs]) and B lymphocytes (regulatory B-lymphocytes [Bregs]) [10,16], which express differential surface receptors and secrete a range of cytokine profiles.

**Figure 1.**
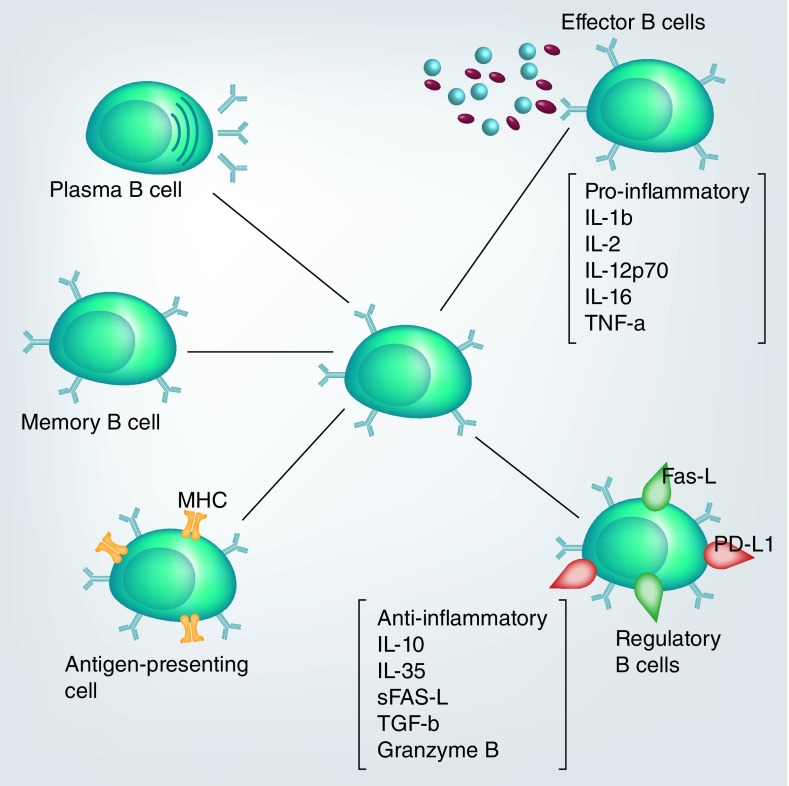
**Different B cell functional response to inflammation.** Stimulation of any of the B cell functions depend on the nature of the pathogenic material, whereas memory B cells are long lasting immunological memory cells that bear specific receptors from previous infection.

Development of Bregs and other B cell subtypes with different immune function (Figure 1) is enhanced by various factors including activated/stimulated cellular pathway, type of stimulant and extracellular concentration of micronutrients [11]. In particular, regulatory function in B cells was first described in experimental autoimmune encephalomyelitis [17]. It was initially thought that the primary function of these Bregs was to maintain the immune environment until Tregs are matured enough to take over the role, as the functions mediated by these cell types (as described by [18]) show them to be alternating, with Bregs regulating early inflammation during experimental autoimmune encephalomyelitis while regulatory T cell frequencies increase toward the late phase of inflammation.

**Figure 2.**
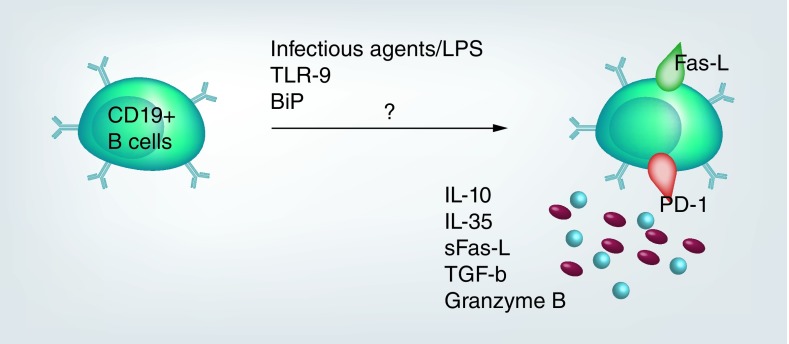
**Biological pathways involved in development of regulatory B cells by various extracellular antigens have not yet been characterized and need further investigations.**

As depicted in Figure 1 and Figure 2, these cells exert their effect through secretion of soluble proteins (blocking specific intracellular pathways) and expression of surface ligand molecules such as Fas-L, FoxP3 and programmed death ligand [10,18], which enhance interaction with cells bearing receptors for those specific ligands and induce apoptosis or programmed death.

Regulatory B cells have been implicated in many inflammatory studies including allograft tolerance, cancer, autoimmune diseases and infection [9,19,20], where they have been shown to inhibit function and proliferation of T helper 1 and T helper 17 cells [21–23]. During autoimmune diseases, these cells increase tolerance of selfantigens, thus preventing destruction of the body's own cells. Similarly, during infection and inflammatory responses, they limit aggressiveness of the immune system and prevent persisting immune responses after clearance of the pathogen. Even though Bregs have not been extensively studied during TB disease, current evidence suggests that B cells with anti-inflammatory properties are present in smaller numbers in the peripheral stream and these decrease drastically during chronic infectious diseases [23–25]. These cells are displayed at higher frequencies in healthy individuals and disappear over time during chronic immune responses, thus leading to immune system imbalance [8,20,25]. Bregs are continuously reported to be dysfunctional during inflammation; furthermore, this is thought to be associated with exacerbation of disease state, especially in autoimmune diseases [26].

Various antigens are known to drive development of Bregs through upregulation of IL-10 secretion. These include BiP, BCG, LPS, infectious agents and TLR-9a [27–29]. In particular, BiP has been shown to resolve inflammation in rheumatoid arthritis by upregulating anti-inflammatory cytokine production by immune cells [30,31]. However, as depicted in Figure 2, the biological pathways involved in the development of these cells by different antigens including BiP remains unknown.

Additionally, functional and phenotypic characterization of these cells is still expanding in the context of different inflammatory conditions and new makers are still being identified that relate to extracellular environment composition during their development. It has been shown by recent studies that apart from IL-10 secretion, these cells are capable of secreting other soluble molecules including sFas-L, TGF-b, granzyme-B and IL-35, which play crucial roles in suppressing inflammation [10,29,32].

### Phenotypically identified subsets of regulatory B cells during inflammation

**Figure 3.**
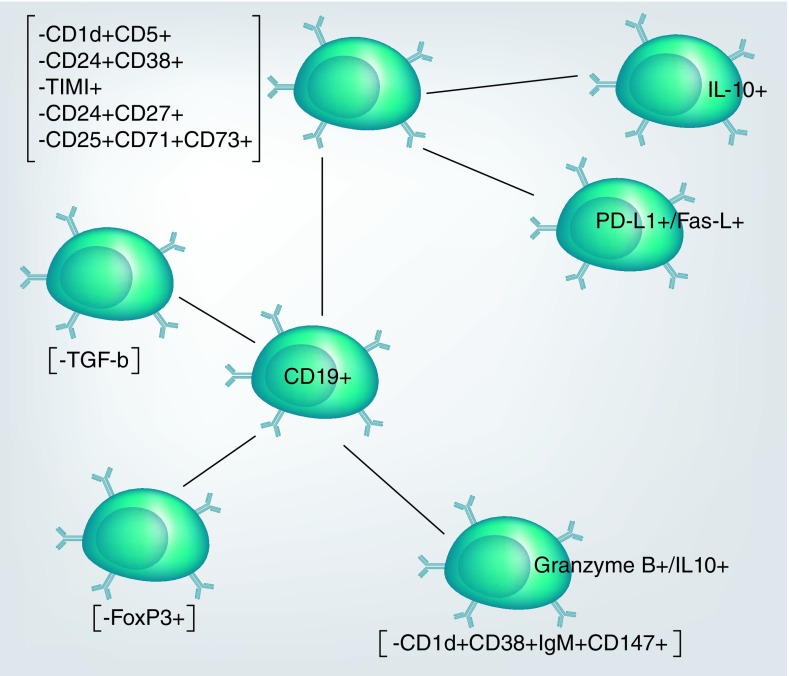
**Circulating CD19^+^ B cells have the potential to develop to regulatory B cells with different regulatory markers depending on their stage of development.** The regulatory trait has been identified within B1 cells, marginal zone B cells and transitional-2 marginal zone B cells.

Identification of Bregs is based on both extracellular surface immunoglobulin expression and cytokine profiles, merely because these properties determine the mode of regulation by these cell types. As postulated in Figure 3 there are four common categories of Bregs and these include IL-10-producing Bregs (commonly known as B*10* cells) that produce IL-10 as major cytokine [33], transforming growth factor-β-producing B cells (B-*TGF-β*) and FoxP3-expressing B cells (B-*FoxP3*) [10]. Additionally, IL-35 is another cytokine secreted by regulatory B cells, either alone or in conjunction with IL-10, to modulate immune responses during inflammation [34]. Several phenotypes have been identified based on extracellular surface immunoglobulin expression within each Bregs category in various studies of both humans and mice.

In humans, the majority of B10 cells are represented by expression of CD19^+^CD24^hi^CD38^hi^, CD1d^hi^CD5^+^, CD19^+^TIM1^+^ on their surface [9,23,33,35]. Similarly, Shen *et al*., (2014) described a subpopulation of IL-35-producing Bregs differentiating from plasma cells expressing the phenotype IgM^+^CD138^hi^TACI^+^CRCX^+^CD1d^+^Tim1^int^ in mice [34]. However, IL-21 has been shown to drive regulatory function in B cells by upregulating expression of granzyme-B in conjunction with IL-10. This subset has been phenotypically identified as CD19^+^CD38^+^CD147^+^CD1d^+^IgM^+^ [36]. Contradicting results in relation to IL-10 were observed in an HIV study whereby GraB^+^ Bregs did not produce IL-10 [36,37]. B cell regulatory function linked with FasL and PD-L1 expression has been described in both inflammatory and healthy state in humans; these appear in varying frequencies in relation to the inflammatory state and were reported to be higher in healthy than in infected individuals [25,29]. CD19^+^FasL^+^ and CD19^+^PD-L1^+^ regulatory subsets secrete IL-10, although IL-10 biosynthesis intracellular pathways are not fully exploited by these B cell subpopulations [25,38].

However, there has been reports that a significant amount of IL-10 production by B cells occurs through involvement of toll-like receptor (TLR) molecules rather than B cell receptors or CD40 involvement [39]. CD1d^hi^CD5^+^ B cells were reported to be inducible for IL-10 secretion in significant amounts through TLR-9 using LPS and intracellularly mediated by MyD88/STAT3 pathway [13,33,39]. FoxP3-expressing B cells in healthy human blood samples were first described in a study by Noh *et al*., (2010) within the CD19^+^CD5^+^ population [40]. These cells appeared in higher frequencies within the CD5^+^ group than in the CD5^-^ group, and they also showed increased apoptotic characteristics than other B cell groups. A subsequent study by Guo *et al*., (2015) described FoxP3-expressing CD19^+^ B cells to be of greater frequencies in healthy individuals than in patients with rheumatoid arthritis (RA) [10].

Although further studies still need to be done on FoxP3^+^ B cells, Guo *et al*., (2015) also compared their frequency with TGF-β CD19^+^ B cells and found no significant difference among the expression frequencies of these groups in both healthy and RA groups [10]. Regulatory function and frequency in B cells and other immune cells is affected by varying antigens in the cellular environment.

### BiP affects immune cell function

The BiP is a 78 kDa heat shock protein naturally occurring in the lumen of the endoplasmic reticulum (ER) and aids in proper peptide folding [41]. Synthesis and expression of this protein has been reported to be upregulated during cellular stress that might be due to glucose starvation or in response to accumulation of unfolded proteins within the cell [42,43]. Its activation/synthesis was initially observed in a study by Munro and Pelham, (1986) where BiP was shown to be expressed in high concentrations in antibody-producing B cells due to high levels of synthesized immunoglobulin inhabiting endoplasmic reticulum than in resting/naive B cells [44]. Gass *et al*., (2002) exploited the unfolded protein response (UPR) in B lymphocytes and found it to be induced during B cell transition to antibody secreting plasma cells, thus suggesting a link between upregulation of this protein with immunoglobulin synthesis [45]. Loss of BiP function in B cells has been associated with inability to secrete functional antibodies, which may ultimately affect opsonization of invading pathogens [46].

**Figure 4.**
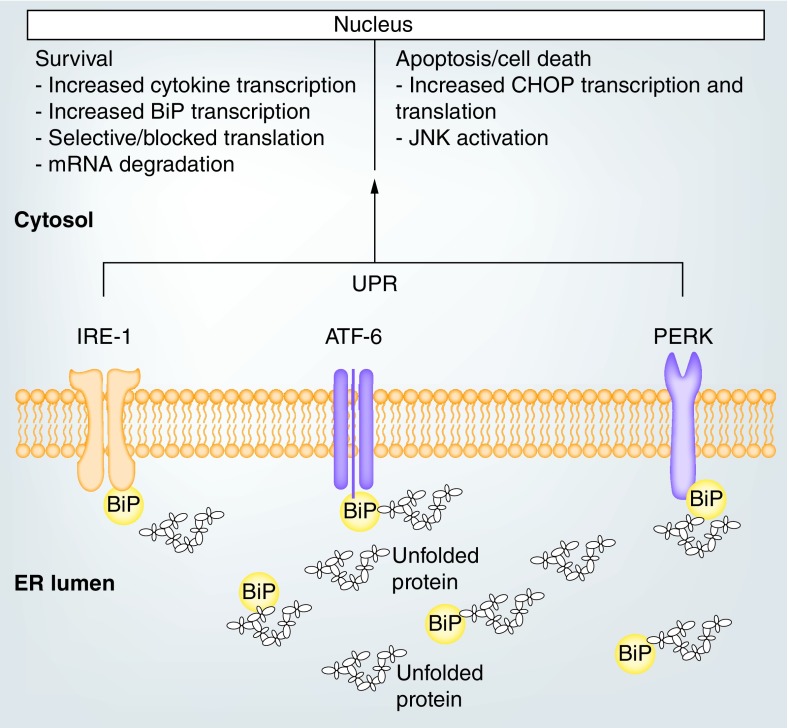
**Unfolded protein response mediated by binding immunoglobulin protein.** Activation of the ER membrane transducers leads to a cascade of kinase phosphorylation that either favors apoptosis through upregulation of CHOP or JNK activation. Similarly, cell survival can be favored by blocking translation and degrading synthesized mRNA. ER: Endoplasmic reticulum; UPR: Unfolded protein response.

In particular, *M. tuberculosis* infection exerts stress on immune cells through secretion of ESAT-6, which affects homeostasis of calcium ions and increases unfolded protein burden in cells due to a metabolic shift. However, it has been implied that extended ER stress can lead to activation of apoptotic pathways in immune cells resulting in skewed immunological responses [8,47]. As illustrated in Figure 4, ER stress activates and upregulates expression of BiP and other ER chaperones to mitigate the conditions affecting cells [42]. These chaperone proteins bind unfolded and partially folded proteins and direct them for proper folding in the ribosomes [48]. They also act as coactivators of three ER membrane signal transducers; PERK, IRE1 and ATF6 (Figure 4), which in turn phosphorylate transcription factors and cytosol kinase involved in translation termination and cytokine synthesis. These mechanisms either promote cell apoptosis or cell survival.

Even though BiP is natively an intracellular protein, it has been reported to be capable of translocating to and being expressed on cell membranes attached to peripheral proteins including both transmembrane and external proteins such as glycosylphosphotidylinositol. Similarly, it can be secreted into the extracellular environment [49] where it acts as an autoantigen and exerts effects on immune cells through binding with surface receptors [50,51]. Over the last few years, its effect has been exploited in different immune cells including macrophages, dendritic cells, monocytes, T cells and B cells. The BiP facilitates differentiation of mature dendritic cells to express an anti-inflammatory phenotype through upregulation of indoleamine-2,3-dioxygenase (IDO) [30] and also stimulates myeloid cells to express an anti-inflammatory phenotype mostly observed in immature dendritic cells [52]. In a study by Corrigall *et al*., (2004) BiP was shown to act on human peripheral blood mononuclear cells to induce anti-inflammatory responses through upregulation of IL-10 [50]. It also affects the expression of CD86 and HLA-DR surface expression on monocytes, which causes these APCs to be unable to activate T cells. The BiP also alters the ability of memory T cells to recall antigens and initiate secondary responses [31].

Immunological effects caused by BiP have ultimately resulted in study in areas relating to cancer, transplantation and autoimmune studies [31,53]. Contradicting results, however, were observed in *Giardia lamblia* infection in murine samples where recombinant BiP extracted from *G lamblia* (rGlBiP) demonstrated a pro-inflammatory response through upregulation of CD80, CD86 and MHC II on mature dendritic cells, leading to increased production of pro-inflammatory cytokines such as IL-6, IL-12 and TNF-α [54]. Another study [41] showed that T cell surface BiP expression can be upregulated by pro-inflammatories such as TNF-α and IL-1β. Additionally, this upregulation was associated with increased proliferation of synoviocyte cells and progressive pathogenesis of RA in synoviocytes. This suggested that T cell surface BiP can modulate pro-inflammatory responses in synoviocytes Interestingly, transfection of the BiP gene into murine and human PBMC samples using a viral vector has been evaluated as having the same anti-inflammatory effects as stimulating arthritis inflammation with synthesised proteins in collagen [55]. Supporting evidence of extracellular BiP binding to immune cells was shown in a study by Tang *et al*., (2016) where BiP induced the development of regulatory B cells in murine samples and worked in synergy with CD40 to suppress proliferation of T cells [29].

### Binding of extracellular BiP with immune cells

The BiP is a member of heat-shock proteins (Hsp70) that has been reported to have immune modulatory functions. Unlike other members of the Hsp70s, which are implicated in induction of pro-inflammatory immune responses, BiP is reported to mostly facilitate anti-inflammatory responses. As a result, this chaperone has been studied in the induction of functional changes in immune cells [50,56,57]. Secreted BiP binds to cell receptors as an autoantigen and causes functional changes. However, pathways involved in these processes are not fully elucidated (Figure 2), although it has been stated that most of the Hsp70 proteins are secreted through passive secretion. Studies on RA and cancer that have identified extracellular BiP observed that it is secreted without its anchor, a four amino acid sequence KDEL, thus suggesting that it is not available as a result of membrane rupture or cell death [50]. In particular, a study in murine collagen induced arthritis revealed an IL-4 dependent effect of BiP on T cell responses that leads to secretion of anticollagen specific cytokines including IL-5 and IL-10 [53]. Even though BiP upregulation is beneficial to immune responses as it activates signal transducers that mediate cytokine and antibody gene expression; similarly, its downregulation has been associated with benefits in cancer suppression that could be a target of choice in studies such as for B cell lymphoma.

Targeting this chaperone could be a promising approach that will aid in the development of new therapeutic procedures that will better control inflammation during diseases [30,52]. Less data have been generated on binding of extracellular BiP onto immune cells since previous studies have focused mainly on monitoring the cytokine profile in immune cell samples in the presence of BiP rather than assessing binding receptors or pathways involved during internalization. However, it has been suggested in a study by Becker *et al*., (2002) that free Hsp70 proteins binds immune cells, such as B cells, through CD40 in an ATPase-dependent manner [58]. Even though this pathway has not been fully studied, BiP binding with immune cells such as B cells was also shown by Tang *et al*., (2016) using fluorescently labeled BiP in murine model [29]. Binding and internalization of this chaperone protein on B cell samples induced upregulation of three populations of Bregs with distinct phenotypes (IL-10^hi^, PDL-1 and FasL), thus highlighting different regulatory mechanisms [29].

### Regulatory function of mature/active regulatory B cells

Initial studies on Bregs implied that these cells were only modulating immune responses until regulatory T cells were mature enough to take over the function. However, further studies elaborated on immune functions played by these cells using murine models and have shown that Bregs exert their function mostly through IL-10 [33,35]. Similar results have been obtained in human models, where both cytokines and cell surface receptors are implicated in the regulatory roles of these cells [59]. Even though their development depends on various factors, including the type of stimulant and the presence of micronutrients to drive immunometabolism, these cells have been shown to inhibit proliferation and function of macrophages, T helper 1 (Th1), and T helper 17 (Th17) cells, while leaving T helper 2 (Th2) function unaffected [22]. In various studies this was confirmed by a decrease in secreted levels of cytokines such as IFN-γ and IL-17, while IL-4 levels appear to not be affected by presence of regulatory B cells [22,23]. Breg importance lies in their maintaining immune balance after inflammatory responses in order to prevent autoimmune diseases progression by suppressing persisting T cell responses [60]. Cell-to-cell immunomodulatory function of Bregs occurs through expression of ligands that target and bind to specific surface receptors expressed by other immune cells and this binding further enhances apoptotic pathways. Such immunomodulatory ligands include PDL-1, which targets PD-1 membrane protein on activated T cells and monocytes; FasL, which stimulates apoptosis of infected macrophages during chronic infections such as TB [25,61,62]; and GITR, which has been shown to be associated with negatively modulating proliferation of regulatory T cells during RA through binding to the GITRligand expressed by these cells [12].

### Immune responses driven by low or high frequencies of Bregs

In allograft and autoimmune diseases, high frequency of Bregs is associated with preventing progression into aggressive stages of inflammation. The functions carried by these cell subsets are highly similar to Tregs; however, these functions are believed to be alternating during inflammation in a sense that Bregs are mostly active during the onset of inflammation whereas Tregs come to action toward the end of inflammation. This notion has been supported in a variety of studies where Bregs were isolated in higher frequencies in healthy individuals but eventually deplete as inflammatory responses progresses [22,35]. The same ‘depletion-reappearing’ trait of these cells has been observed in studies focusing on different inflammatory responses using both murine and human models [8,22,25,35]. Recently, it has been observed in human samples studying B cell immune responses during *M. tuberculosis* infection. This was believed to play a part in disease progression as their numbers vanished during active TB disease but, upon treatment, Bregs reappeared to a frequency equivalent to healthy individuals [62]. Expression of regulatory traits in B cells has been described in varying frequencies depending on B cell development stage, location and type of inflammation. Highlighted in Table 1 are the recently reported frequencies of different Breg populations in uninfected or healthy individuals and these range from 0.1% to as high as 12% of the total described B cell population. However, *in vitro* these frequency percentages could be stimulated to increase using different stimulants among which are LPS, TLR-9 agonist and BGC [24,38,59].

**Table 1.** **Defined regulatory B cell population frequencies in healthy human individuals expressing different surface markers and exhibiting diverse regulatory mechanisms.**

**Phenotype**	**Species**	**Percentage (%)**	**Mechanism of suppression**	**[Ref.]**
CD24^hi^CD27^+^	Human, PBMC	0.6	IL-10	[63]

CD24^+^CD38^+^ CD24^hi^CD38^-^ CD24^hi^CD38^int^ CD24^hi^CD38^hi^	Human	3.92 3.71 12.6	IL-10	[59]

CD5^+^CD1d^+^IL10^+^	Human, PBMC	0.24 ± 0.05	IL1-0	[64]

CD19^+^IgM^+^CD38^+^ – FasL^+^ – PD1^+^	Human, PBMC	0.1–1.6	FasL	[25]

CD19^+^IgM^-^CD38^+^ – FasL^+^ – PD1^+^	Human, PBMC	0.1–1.5	FasL	[25]

CD19^+^PDL1^hi^ CD19^+^CD138^+^CD27^+^	Human, spleen Human, PBMC	5.2 2.99	PDL1 Il-10	[18] [24]

Presence of Bregs and high secretion levels of IL-10 from these cells modulates differentiation of certain T cell subsets expressing CD4^+^CD25^-^ T cells to Tregs expressing FoxP3, which further mediate regulatory functions during inflammation [22]. *In vitro* work has shown that high levels of IL-10 are linked to impaired secretion and activity of certain pro-inflammatory cytokines such as TNF-α, IL-12, IL1-β and IL-17 [22,23,65,66]; these are required during early inflammation to initiate and activate adaptive responses by recruiting T- and B cells. However, in the context of *M. tuberculosis* infection, engagement of adaptive responses has been speculated to favor TB disease progression through the formation of granulomas that later become replication sites for the intracellular pathogen, leading to more cells being infected within the structure as bacterial numbers grow and infected cells rupture through cellular necrosis and pyroptosis.

### Regulatory B cells as agents in therapeutic interventions

Inflammatory responses directed to intracellular pathogens such as *M. tuberculosis* face challenges in directly eliminating the pathogen from inside the cell [67]. This phenomenon results as pathogens such as *M. tuberculosis* have evolved a way to suppress destruction by proteolytic enzymes inside the phagosomes [68,69]; additionally, antibodies and cytokines secreted toward this pathogen cannot penetrate cellular membranes to reach it, thus resulting in established infection and disease progression [70]. Even though formation of granulomatous structures during TB helps in containing the pathogen and preventing early spread [71], the pathogen may multiply to higher numbers and progress to active disease [7,71]. Immune cell activity within the granuloma structure and upregulation of necrotic cytokines and perforins lead to destruction of infected cells by necrosis, which in turn leads to the release of more pathogens to infect other cells [72,73]. Comparatively, Bregs have been suggested to induce apoptosis of infected cells by affecting cellular metabolism – which in turn affects homeostasis of calcium – reactive oxygen species and nitric oxide, thus affecting survival of the intracellular pathogen [72,74]. This prevents presentation of antigens and activation of effector T cells, thus limiting activation of adaptive immune responses [31,75]. Stimulating development or maintaining frequency of these regulatory cells can present more benefits in host-directed immunotherapies and control of intracellular pathogens like *M. tuberculosis*.

## Conclusion & future perspective

### Regulatory characteristic induction by BiP in B cells

Little data are currently available on the induction of human regulatory B cells using BiP, indicating that more work still needs to be done to understand the pathways involved in the development of these cell types. Previous reports have shown that BiP has anti-inflammatory properties through induction of regulatory cells and this has been evaluated in different inflammatory studies with successful results [31,57]. Apart from inducing immune regulatory functions, its activity in immunoglobulin folding results in better antibody secretion which in turn facilitates better control of infectious pathogens [76]. Its biological nature gives it an additional advantage by limiting nonspecific inflammatory responses that may end up causing immuno-pathogenesis. The availability of this molecule in extracellular circulation and its autoantigen properties facilitate secretion of autoantibodies against it. This also shows an additional advantage since immune cells will efficiently opsonize and internalize it, resulting in shorter time required for development of regulatory traits. Induction of these traits in B cells using BiP in a murine model study and its implication in inducing regulatory traits in T cells in human model studies have paved the way for, and indicated a high possibility for, Breg induction by this antigen in human settings [29]. More studies must be conducted in the context of *M. tuberculosis* infection and BiP behavior.

**Executive summary**

- Antigen presenting cells (APCs) opsonize and internalize invading *M. tuberculosis* particles; however, this pathogen is able to reside and multiply within these cells leading to granulomatous structure formation.
- B cells can differentiate into a variety of subsets, which includes effector B cells, plasma, memory and regulatory cells.
- During chronic diseases, including tuberculosis, B cells are shown to be dysfunctional in blood circulation and *M. tuberculosis* infection takes advantage of this immune imbalance.
- Regulatory functions mediated by B cells was described during experimental autoimmune encephalomyelitis and shown to resolve disease progression.
- Apart from IL-10 secretion, these cells express surface markers such as sFas-L, PD-L1 and FoxP3.
- There are four groups of regulatory B cells which are grouped based on their mode of action; IL-10 producing B cells, TGF-β secreting B cells, IL-35 secreting B cells and FoxP3 expressing B cells.
- Regulatory B cells are known to be inducible by various factors such as BiP, BCG, LPS, infectious agents and TLR-9a.
- Binding immunoglobulin protein (BiP) resides in the endoplasmic reticulum and facilitate proper protein folding; its activity is induced during cellular stress conditions posed onto cells by accumulation of unfolded proteins among others.
- Its activation results from the unfolded protein response pathway, which is thought to be a survival mechanism that T cells use during such conditions; however, prolonged cell stress can lead to induction of apoptotic pathways.
- Unfolded protein response is also mediated by three endoplasmic reticulum membrane-bound transducers (PERK, IRE1 and ATF6) that act as coactivators during this pathway and their activity determines between cell survival or cell apoptosis.
- The BiP can escape intracellular environment and act as autoantigen in the extracellular matrix and its effect has been observed in various cell types including macrophages, dendritic cells, monocytes T and B cells.
- Its presence in the extracellular matrix has been shown to induce regulatory traits on immune cells and further resolve inflammation.
- Regulatory B cells are known to inhibit proliferation and functions mediated by macrophages, T helper 1 and T helper 17 cells, while leaving T helper 2 function unaffected.
- High frequencies of regulatory B cells are associated with preventing disease progression.
- Regulatory B cells disappearance during chronic diseases is thought to play part in disease progression particularly during TB.
- Ability of BiP to modulate immunological responses and drive anti-inflammatory properties has set a promising research platform in chronic inflammation.
- Its secretion is suggested to be passive in conjunction with newly synthesized proteins as it was isolated in the extracellular matrix during rheumatoid arthritis without its membrane anchor sequence.

## References

[1] Brennan MA, Cookson BT (2000). Salmonella induces macrophage death by caspase-1-dependent necrosis. *Mol. Microbiol.*.

[2] Athman JJ, Sande OJ, Groft SG (2017). Mycobacterium tuberculosis membrane vesicles inhibit T cell activation. *J. Immunol.*.

[3] Raghuvanshi S, Sharma P, Singh S, Van Kaer L, Das G (2010). Mycobacterium tuberculosis evades host immunity by recruiting mesenchymal stem cells. *Proc. Natl Acad. Sci.*.

[4] Costello AM, Kumar A, Narayan V (1992). Does antibody to mycobacterial antigens, including lipoarabinomannan, limit dissemination in childhood tuberculosis?. *Trans. R. Soc. Trop. Med. Hyg.*.

[5] de Valliere S, Abate G, Blazevic A, Heuertz RM, Hoft DF (2005). Enhancement of innate and cell-mediated immunity by antimycobacterial antibodies. *Infect. Immun.*.

[6] Davis JM, Clay H, Lewis JL, Ghori N, Herbomel P, Ramakrishnan L (2002). Real-time visualization of mycobacterium-macrophage interactions leading to initiation of granuloma formation in zebrafish embryos. *Immunity*.

[7] Davis JM, Ramakrishnan L (2009). The role of the granuloma in expansion and dissemination of early tuberculous infection. *Cell*.

[8] Joosten SA, van Meijgaarden KE, del Nonno F (2016). Patients with tuberculosis have a dysfunctional circulating B cell compartment, which normalizes following successful treatment. *PLoS Pathog.*.

[9] Ma L, Liu B, Jiang Z, Jiang Y (2014). Reduced numbers of regulatory B cells are negatively correlated with disease activity in patients with new-onset rheumatoid arthritis. *Clin. Rheumatol.*.

[10] Guo Y, Zhang X, Qin M, Wang X (2015). Changes in peripheral CD19(+)Foxp3(+) and CD19(+)TGFβ(+) regulatory B cell populations in rheumatoid arthritis patients with interstitial lung disease. *J. Thorac. Dis.*.

[11] Holan V, Zajicova A, Javorkova E (2014). Distinct cytokines balance the development of regulatory T cells and interleukin-10-producing regulatory B cells. *Immunology*.

[12] Ray A, Basu S, Williams CB, Salzman NH, Dittel BN (2012). A novel IL-10-independent regulatory role for B cells in suppressing autoimmunity by maintenance of regulatory T cells via GITR ligand. *J. Immunol.*.

[13] Bénard A, Sakwa I, Schierloh P (2018). B cells producing type I IFN modulate macrophage polarization in tuberculosis. *Am. J. Respir. Crit. Care Med.*.

[14] Cooper MA, Fehniger TA, Turner SC (2001). Human natural killer cells: a unique innate immunoregulatory role for the CD56(bright) subset. *Blood*.

[15] Serafini P, Mgebroff S, Noonan K, Borrello I (2008). Myeloid-derived suppressor cells promote cross-tolerance in B cell lymphoma by expanding regulatory T cells. *Cancer Res.*.

[16] Mahic M, Yaqub S, Johansson CC, Taskén K, Aandahl EM (2006). FOXP3+CD4+CD25+ adaptive regulatory T cells express cyclooxygenase-2 and suppress effector T cells by a prostaglandin e2-dependent mechanism. *J. Immunol.*.

[17] Wolf SD, Dittel BN, Hardardottir F, Janeway CA (1996). Experimental autoimmune encephalomyelitis induction in genetically B cell-deficient mice. *J. Exp. Med.*.

[18] Khan AR, Hams E, Floudas A, Sparwasser T, Weaver CT, Fallon PG (2015). PD-L1hi B cells are critical regulators of humoral immunity. *Nat. Commun.*.

[19] Lee KM, Stott RT, Zhao G (2014). TGF-β-producing regulatory B cells induce regulatory T cells and promote transplantation tolerance: immunomodulation. *Eur. J. Immunol.*.

[20] Li W, Tian X, Lu X (2016). Significant decrease in peripheral regulatory B cells is an immunopathogenic feature of dermatomyositis. *Sci. Rep.*.

[21] Carter NA, Vasconcellos R, Rosser EC (2011). Mice lacking endogenous IL-10-producing regulatory B cells develop exacerbated disease and present with an increased frequency of Th1/Th17 but a decrease in regulatory T cells. *J. Immunol.*.

[22] Flores-Borja F, Bosma A, Ng D (2013). CD19+CD24hiCD38hi B cells maintain regulatory T cells while limiting TH1 and TH17 differentiation. *Sci. Transl. Med.*.

[23] Zhang M, Zheng X, Zhang J (2012). CD19+CD1d+CD5+ B cell frequencies are increased in patients with tuberculosis and suppress Th17 responses. *Cell. Immunol.*.

[24] du Plessis WJ, Keyser A, Walzl G, Loxton AG (2016). Phenotypic analysis of peripheral B cell populations during *Mycobacterium tuberculosis* infection and disease. *J. Inflamm. (Lond.)*.

[25] van Rensburg IC, Kleynhans L, Keyser A, Walzl G, Loxton AG (2017). B cells with a FasL expressing regulatory phenotype are induced following successful anti-tuberculosis treatment: TB treatment induces FasL regulatory B cells. *Immun. Inflamm. Dis.*.

[26] Fillatreau S, Sweenie CH, McGeachy MJ, Gray D, Anderton SM (2002). B cells regulate autoimmunity by provision of IL-10. *Nat. Immunol.*.

[27] du Plessis WJ, Kleynhans L, du Plessis N (2016). The functional response of B cells to antigenic stimulation: a preliminary report of latent tuberculosis. Wilkinson KA (Ed.). *PLoS ONE*.

[28] Lenert P, Brummel R, Field EH, Ashman RF (2005). TL R-9 activation of marginal zone B cells in lupus mice regulates immunity through increased IL-10 production. *J. Clin. Immunol.*.

[29] Tang Y, Jiang Q, Ou Y (2016). BIP induces mice CD19(hi) regulatory B cells producing IL-10 and highly expressing PD-L1, FasL. *Mol. Immunol.*.

[30] Corrigall VM, Vittecoq O, Panayi GS (2009). Binding immunoglobulin protein-treated peripheral blood monocyte-derived dendritic cells are refractory to maturation and induce regulatory T cell development. *Immunology*.

[31] Yoshida K, Ochiai A, Matsuno H, Panayi GS, Corrigall VM (2011). Binding immunoglobulin protein resolves rheumatoid synovitis: a xenogeneic study using rheumatoid arthritis synovial membrane transplants in SCID mice. *Arthritis Res. Ther.*.

[32] Zhang Y, Morgan R, Chen C (2016). Mammary-tumor-educated B cells acquire LAP/TGF-β and PD-L1 expression and suppress anti-tumor immune responses. *Int. Immunol.*.

[33] Yanaba K, Bouaziz J-D, Matsushita T, Tsubata T, Tedder TF (2009). The development and function of regulatory B cells expressing IL-10 (B10 cells) requires antigen receptor diversity and TLR signals. *J. Immunol.*.

[34] Shen P, Roch T, Lampropoulou V, O'Connor RA, Stervbo U, Hilgenberg E (2014). IL-35-producing B cells are critical regulators of immunity during autoimmune and infectious diseases. *Nature*.

[35] Matsushita T, Yanaba K, Bouaziz J-D, Fujimoto M, Tedder TF (2008). Regulatory B cells inhibit EAE initiation in mice while other B cells promote disease progression. *J. Clin. Invest.*.

[36] Lindner S, Dahlke K, Sontheimer K (2013). Interleukin 21-induced granzyme B-expressing B cells infiltrate tumors and regulate T cells. *Cancer Res.*.

[37] Kaltenmeier C, Gawanbacht A, Beyer T (2015). CD4^+^ T cell-derived IL-21 and deprivation of CD40 signaling favor the *In vivo* development of granzyme B–expressing regulatory B cells in HIV patients. *J. Immunol.*.

[38] van Rensburg IC, Loxton AG (2018). Killer (FASL regulatory) B cells are present during latent TB and are induced by BCG stimulation in participants with and without latent tuberculosis. *Tuberculosis*.

[39] Liu B-S, Cao Y, Huizinga TW, Hafler DA, Toes REM (2014). TLR-mediated STAT3 and ERK activation controls IL-10 secretion by human B cells. *Eur. J. Immunol.*.

[40] Noh J, Choi WS, Noh G, Lee JH (2010). Presence of *Foxp3* -expressing CD19(+)CD5(+) B cells in human peripheral blood mononuclear cells: human CD19(+)CD5(+) *Foxp3* (+) regulatory B cell (Breg). *Immune Netw.*.

[41] Yoo S-A, You S, Yoon H-J (2012). A novel pathogenic role of the ER chaperone GRP78/BiP in rheumatoid arthritis. *J. Exp. Med.*.

[42] Choi H-H, Shin D-M, Kang G (2010). Endoplasmic reticulum stress response is involved in *Mycobacterium tuberculosis* protein ESAT-6-mediated apoptosis. *FEBS Lett.*.

[43] Lee AS (2005). The ER chaperone and signaling regulator GRP78/BiP as a monitor of endoplasmic reticulum stress. *Methods*.

[44] Munro S, Pelham HR (1986). An Hsp70-like protein in the ER: identity with the 78 kd glucose-regulated protein and immunoglobulin heavy chain binding protein. *Cell*.

[45] Gass JN, Gifford NM, Brewer JW (2002). Activation of an unfolded protein response during differentiation of antibody-secreting B cells. *J. Biol. Chem.*.

[46] Hu C-CA, Dougan SK, Winter SV, Paton AW, Paton JC, Ploegh HL (2009). Subtilase cytotoxin cleaves newly synthesized BiP and blocks antibody secretion in B lymphocytes. *J. Exp. Med.*.

[47] Li J, Lee B, Lee AS (2006). Endoplasmic reticulum stress-induced apoptosis multiple pathways and activation of p53-up-regulated modulator of apoptosis (puma) and noxa BYp53. *J. Biol. Chem.*.

[48] Maddalo D, Neeb A, Jehle K (2012). A peptidic unconjugated GRP78/BiP ligand modulates the unfolded protein response and induces prostate cancer cell death. *PLoS ONE*.

[49] Tsai Y-L, Zhang Y, Tseng C-C, Stanciauskas R, Pinaud F, Lee AS (2015). Characterization and mechanism of stress-induced translocation of 78-kilodalton glucose-regulated protein (GRP78) to the cell surface. *J. Biol. Chem.*.

[50] Corrigall VM, Bodman-Smith MD, Brunst M, Cornell H, Panayi GS (2004). Inhibition of antigen-presenting cell function and stimulation of human peripheral blood mononuclear cells to express an antiinflammatory cytokine profile by the stress protein BiP: relevance to the treatment of inflammatory arthritis. *Arthritis Rheum.*.

[51] Zhang Y, Liu R, Ni M, Gill P, Lee AS (2010). Cell surface relocalization of the endoplasmic reticulum chaperone and unfolded protein response regulator GRP78/BiP. *J. Biol. Chem.*.

[52] Yang M, Zhang F, Qin K (2016). Glucose-regulated protein 78-induced myeloid antigen-presenting cells maintained tolerogenic signature upon LPS stimulation. *Front. Immunol.*.

[53] Brownlie RJ, Myers LK, Wooley PH (2006). Treatment of murine collagen-induced arthritis by the stress protein BiP via interleukin-4–producing regulatory T cells: a novel function for an ancient protein. *Arthritis Rheum.*.

[54] Lee H-Y, Kim J, Noh HJ, Kim H-P, Park S-J (2014). *Giardia lamblia* binding immunoglobulin protein triggers maturation of dendritic cells via activation of TLR4-MyD88-p38 and ERK1/2 MAPKs. *Parasite Immunol.*.

[55] Shields AM, Klavinskis LS, Antoniou M (2015). Systemic gene transfer of binding immunoglobulin protein (BiP) prevents disease progression in murine collagen-induced arthritis: lentiviral delivered BiP reduces inflammation. *Clin. Exp. Immunol.*.

[56] Bläss S, Union A, Raymackers J (2001). The stress protein BiP is overexpressed and is a major B and T cell target in rheumatoid arthritis. *Arthritis Rheum.*.

[57] Bodman-Smith MD, Corrigall VM, Kemeny DM, Panayi GS (2003). BiP, a putative autoantigen in rheumatoid arthritis, stimulates IL-10-producing CD8-positive T cells from normal individuals. *Rheumatol. Oxf. Engl.*.

[58] Becker T, Hartl F-U, Wieland F (2002). CD40, an extracellular receptor for binding and uptake of Hsp70–peptide complexes. *J. Cell. Biol.*.

[59] Blair PA, Noreña LY, Flores-Borja F (2010). CD19+CD24hiCD38hi B cells exhibit regulatory capacity in healthy individuals but are functionally impaired in systemic lupus erythematosus patients. *Immunity*.

[60] Yanaba K, Bouaziz J-D, Haas KM, Poe JC, Fujimoto M, Tedder TF (2008). A regulatory B cell subset with a unique CD1dhiCD5+ phenotype controls T cell-dependent inflammatory responses. *Immunity*.

[61] Oddo M, Renno T, Attinger A, Bakker T, MacDonald HR, Meylan PR (1998). Fas ligand-induced apoptosis of infected human macrophages reduces the viability of intracellular Mycobacterium tuberculosis. *J. Immunol.*.

[62] van Rensburg IC, Wagman C, Stanley K (2017). Successful TB treatment induces B cells expressing FASL and IL5RA mRNA. *Oncotarget*.

[63] Iwata Y, Matsushita T, Horikawa M (2011). Characterization of a rare IL-10-competent B cell subset in humans that parallels mouse regulatory B10 cells. *Blood*.

[64] Chen Y, Li C, Lu Y (2017). IL-10-producing CD1dhiCD5+ regulatory B cells may play a critical role in modulating immune homeostasis in silicosis patients. *Front. Immunol.*.

[65] Fiorentino DF, Zlotnik A, Vieira P (1991). IL-10 acts on the antigen-presenting cell to inhibit cytokine production by Th1 cells. *J. Immunol.*.

[66] Horikawa M, Weimer ET, DiLillo DJ (2013). Regulatory B cell (B10 cell) expansion during listeria infection governs innate and cellular immune responses in mice. *J. Immunol.*.

[67] Winslow GM, Yager E, Shilo K, Volk E, Reilly A, Chu FK (2000). Antibody-mediated elimination of the obligate intracellular bacterial pathogen ehrlichia chaffeensis during active infection. *Infect. Immun.*.

[68] Russell DG (2001). *Mycobacterium tuberculosis:* here today, and here tomorrow. *Nat. Rev. Mol. Cell. Biol.*.

[69] Woo M, Wood C, Kwon D, Park K-HP, Fejer G, Delorme V (2018). *Mycobacterium tuberculosis* infection and innate responses in a new model of lung alveolar macrophages. *Front. Immunol.*.

[70] Achkar JM, Chan J, Casadevall A (2015). B cells and antibodies in the defense against mycobacterium tuberculosis infection. *Immunol. Rev.*.

[71] Ehlers S, Schaible UE (2013). The granuloma in tuberculosis: dynamics of a host–pathogen collusion. *Front. Immunol.*.

[72] Divangahi M, Chen M, Gan H (2009). Mycobacterium tuberculosis evades macrophage defenses by inhibiting plasma membrane repair. *Nat. Immunol.*.

[73] Chen M, Gan H, Remold HG (2006). A mechanism of virulence: virulent mycobacterium tuberculosis strain H37Rv, but not attenuated H37Ra, causes significant mitochondrial inner membrane disruption in macrophages leading to necrosis. *J. Immunol.*.

[74] Jang K-J, Mano H, Aoki K (2015). Mitochondrial function provides instructive signals for activation-induced B cell fates. *Nat. Commun.*.

[75] Axelsson-Robertson R, Ju JH, Kim H-Y, Zumla A, Maeurer M (2015). *Mycobacterium tuberculosis*-specific and MHC Class I-restricted CD8+ T cells exhibit a stem cell precursor-like phenotype in patients with active pulmonary tuberculosis. *Int. J. Infect. Dis.*.

[76] Fritz JM, Weaver TE (2014). The BiP cochaperone ERdj4 is required for B cell development and function.. *PLoS ONE*.

